# XVII International AIDS Conference: From Evidence to Action - Introduction

**DOI:** 10.1186/1758-2652-12-S1-S1

**Published:** 2009-10-06

**Authors:** Rodney Kort

**Affiliations:** 1 Kort Consulting, Toronto, M4Y 2T6, Canada

## Introduction

For over 25 years, International AIDS Conferences have played a key role in the response to AIDS: by providing the research community with opportunities to share important scientific advances, by profiling both successes and failures in the response to AIDS, and by offering a unique platform for people working professionally in the HIV field to address critical issues before a global audience.

Whether reporting on scientific breakthroughs, raising the profile of social justice issues, or highlighting gaps in the response, the conference has acted as a benchmark for the HIV field to assess progress and address both new and ongoing challenges to prevent, treat and control HIV.

The XVII International AIDS Conference (AIDS 2008), held 3 - 8 August 2008 in Mexico City, was an opportunity for HIV professionals to explore the latest scientific research, best practices and programmatic experience in the global response to AIDS. It also provided participants with a variety of formats for structured dialogue and debate on a broad range of HIV issues, including how to use the evidence base, knowledge and skills of those working in HIV to influence political leaders to implement proven interventions, increase their commitment to the response to HIV, and be more accountable for their national and international commitments. AIDS 2008 organizers identified key issues to be discussed in advance of the conference, including:

• Health systems strengthening, service models, integration with TB and sexual and reproductive health services, co-infections (such as malaria and viral hepatitis), maternal health, long-term care, mental health, palliative care, drug resistance, harm reduction, primary health care, and hepatitis C

• Specific regional issues (e.g. stigma; setting regional policy agendas; using what is gained and expanding to other regions)

• Synergy of treatment and prevention; treatment as a tool for prevention (reducing viral load and infectivity)

• Respect and promotion of human rights and gender equality as a framework for all aspects of the response

While AIDS 2008 was notable for the diversity and scope of its programme, this supplement focuses on content areas in which important new research, other evidence or lessons learned were presented, as well as on issues that generated significant discussion, debate and controversy that are likely to have a substantial impact on the global response in the coming months and years. The supplement is adapted from a report released in English and Spanish several months following the conference: The AIDS 2008 Impact Report: Evidence to Action, available at http://www.iasociety.org/Default.aspx?pageId=147.

## Methods

The International AIDS Society (IAS) recruited a team of writers with content expertise relevant to the scientific, leadership and community programmes of the conference to draft a post-conference report. Writers attended conference presentations as well as satellite sessions, pre-conference meetings, the Global Village, the youth programme and other events and activities outside of the formal conference programme.

The writers confirmed leads for relevant programme areas and scientific programme tracks prior to the conference, and met for daily briefings during the conference to discuss emerging issues, report on their findings and informal discussions with conference delegates, and adjust their itineraries as required. The writers met immediately following the conference to confirm the conference highlights and sessions that, based on their analysis and final rapporteur sessions, would likely have the most significant impact on the HIV field in the future and should therefore be reflected in the report.

Each writer then submitted a report that was reviewed, edited, and combined into a draft report which was then peer-reviewed by experts in the field. The final English language report, The AIDS 2008 Impact Report: Evidence to Action was released in December 2008 [1]. A Spanish translation was released in March 2009.

## Discussion

AIDS 2008 demonstrated both the enormous progress and outstanding challenges in the global response to AIDS as the 2010 deadline for universal access nears. As the first International AIDS Conference to be held in Latin America, it served as a platform for a number of commitments made by political leaders across the region, both prior to and during the conference. From the commitment of the host country to fight homophobia and other barriers to meeting universal access targets within Mexico, to the regional commitments on HIV-related health, education and treatment issues, AIDS 2008 has had - and continues to have - an impact well beyond the five days of the conference itself.

The focus in this supplement is on profiling the presentations, discussion and debate that are likely to have the most significant impact on the epidemic in advancing an evidence-based response to HIV/AIDS. The AIDS 2008 Impact Report: Evidence to Action reviews highlights from the conference programmes (Science, Community and Leadership), programme activities, pre-conference events and other activities to assess their impact on the field.

The purpose of the AIDS 2008 Impact Report and this supplement is to assist stakeholders in implementing the evidence-based policies and programmes required to achieve universal access to HIV prevention, treatment, care and support. Although it is always difficult to determine with precision the impact of a conference as large and thematically diverse as the International AIDS Conference, this report reflects the best analysis of the writers and reviewers who attended the many sessions, satellites and affiliated events of AIDS 2008. Session codes are included in each figure and citation to facilitate searches for the source presentation on either the JIAS Abstract Database http://www.jiasociety.org/ or IAS Abstract Database http://www.iasociety.org.

### Organization of the supplement

The supplement is contains six articles, each of which includes an analysis of the implications and potential impact of the major developments reported at AIDS 2008 in the areas of research, programme development, policy and advocacy.

#### 1. Epidemiology

A brief review of global epidemiology and current challenges in assessing the incidence and prevalence of HIV, focusing on HIV surveillance and other strategic health information related to key populations [2].

#### 2. Basic science

An analysis of new evidence presented in Track A of the Scientific Programme, as well as in related sessions, activities and affiliated events [3].

#### 3. Clinical and biomedical prevention science

An analysis of new evidence presented in Tracks C and D of the Scientific Programme, as well as in related sessions, activities and affiliated events [4].

#### 4. Social, economic and political science and policy

An analysis of new evidence presented in Tracks D and E of the Scientific Programme, as well as in other related sessions, activities and affiliated events [5].

#### 5. Regional focus

A review of the current response to the epidemic in the six regions, as well as a discussion of major lessons learned presented at AIDS 2008, and an assessment of the challenges, opportunities and future policy and advocacy priorities within each of these regions:

▪ Asia and the Pacific Islands

▪ Eastern Europe and Central Asia

▪ Middle East and North Africa

▪ Sub-Saharan Africa

▪ Caribbean

▪ Latin America

Information presented in this article is drawn primarily from the Leadership and Community Programmes, as well as related programme activities and affiliated events [6].

#### 6. AIDS 2008 and the global response: tracking progress and strengthening accountability

A review of major discussions at AIDS 2008 regarding how to improve efforts to track progress and strengthen accountability in the global response to AIDS, focusing particularly on strategies to enhance the role of the conference as an accountability mechanism [7].

Specific programmes and initiatives are included in each article to illustrate both successes and challenges in research, programme rollout, policy and advocacy.

## Conclusion

The most important takeaway message from the conference is that a combination of insufficient resources, unmet commitments and structural barriers are preventing the full implementation of evidence-based prevention, treatment and care interventions. AIDS 2008, perhaps more than any previous International AIDS Conference, brought a renewed focus on the legal and human rights issues faced by vulnerable and most at risk populations both in generalized and concentrated or low-level epidemics.

The evidence and experience from the conference were unequivocal in their message: until leaders in both government and civil society are able to separate personal morality and political expediency from the evidence-based interventions required to halt and begin to reverse this epidemic, universal access targets will not be met.

The IAS hopes this supplement will be a useful resource for HIV professionals working in every sector of the response to AIDS, and that it will become a powerful tool used to strengthen the response to HIV/AIDS around the globe.

## Competing interests

Rodney Kort is an independent consultant contracted by the International AIDS Society for the purpose of preparing the AIDS 2008 Impact Report and adapting it for publication in the Journal of the International AIDS Society.
